# Stimulator of Interferon Genes Deficiency in Acute Exacerbation of Idiopathic Pulmonary Fibrosis

**DOI:** 10.3389/fimmu.2017.01756

**Published:** 2017-12-11

**Authors:** Hui Qiu, Dong Weng, Tao Chen, Li Shen, Shan-Shan Chen, Ya-Ru Wei, Qin Wu, Meng-Meng Zhao, Qiu-Hong Li, Yang Hu, Yuan Zhang, Ying Zhou, Yi-Liang Su, Fen Zhang, Li-Qin Lu, Nian-Yu Zhou, Sen-Lin Li, Le-Le Zhang, Chen Wang, Hui-Ping Li

**Affiliations:** ^1^Department of Respiratory Medicine, Shanghai Pulmonary Hospital, School of Medicine, Tongji University, Shanghai, China; ^2^School of Medicine, Suzhou University, Suzhou, China; ^3^State Key Laboratory of Cell Biology, Institute of Biochemistry and Cell Biology, Shanghai Institutes for Biological Sciences, Chinese Academy of Sciences, Shanghai, China; ^4^School of Life Science and Technology, China Pharmaceutical University, Nanjing, China

**Keywords:** stimulator of interferon genes, acute exacerbation of idiopathic pulmonary fibrosis, endoplasmic reticulum stress, viral infection, apoptosis

## Abstract

The stimulator of interferon genes (STING) is a key adaptor protein mediating innate immune defense against DNA viruses. To investigate the role of STING in acute exacerbation of idiopathic pulmonary fibrosis (AE-IPF), we isolated primary peripheral blood mononuclear cells (PBMCs) from patients and healthy controls (HCs). Raw264.7 and A549 cells were infected with herpes simplex virus type 1 (HSV-1). Mice with bleomycin-induced lung fibrosis were infected with HSV-1 to stimulate acute exacerbation of the lung fibrosis. Global gene expression profiling revealed a substantial downregulation of interferon-regulated genes (downstream of STING) in the AE-IPF group compared with the HC and stable IPF groups. The PBMCs of the AE-IPF group showed significantly reduced STING protein levels, increased levels of endoplasmic reticulum (ER) stress markers, and elevated apoptosis. HSV-1 infection decreased STING expression and stimulated the ER stress pathways in Raw264.7 and A549 cells in a time- and dose-dependent manner. HSV-1 infection exacerbated the bleomycin-induced lung injury in mice. In the primary bone marrow-derived macrophages of mice treated with bleomycin and HSV-1, STING protein expression was substantially reduced; ER stress was stimulated. Tauroursodeoxycholic acid, a known inhibitor of ER stress, partially reversed those HSV-1-mediated adverse effects in mice with bleomycin-induced lung injury. STING levels in PBMCs increased after treatment in patients showing improvement but remained at low levels in patients with deterioration. Viral infection may trigger ER stress, resulting in STING deficiency and AE-IPF onset.

## Introduction

Acute exacerbation of idiopathic pulmonary fibrosis (AE-IPF) is the leading cause of death in patients with IPF (1, 2). Although the exact etiology of AE-IPF remains unclear, recent studies suggest that viral infection may contribute to the pathogenesis of AE-IPF (2). Wootton et al. have identified respiratory viral infection in some patients with AE-IPF *via* pan-viral arrays and polymerase chain reaction (3). Stimulator of interferon genes (STING), also known as MITA or MPYS, is encoded by the TMEM173 gene and plays a key role in viral DNA-sensing pathways by regulating the expression of numerous host defense genes, including type I interferons (IFNs) and pro-inflammatory cytokines (4–7). TMEM173 gene mutation has been found in patients with severe pulmonary fibrosis, suggesting that STING may be involved in AE-IPF (8). The molecular mechanism underlying the role of STING in AE-IPF is unknown.

Stimulator of interferon genes is an endoplasmic reticulum (ER) resident protein. Thus, ER functional disorder may damage STING, which may consequently compromise the host defense mechanism against virus. A wide range of pathological stimuli including virus can cause unfolded protein accumulation in the ER, triggering unfolded protein response (UPR). Early-stage UPR restores ER homeostasis, but prolonged or severe UPR can lead to ER stress, inducing cell death and damaging host immune response (9, 10). Herpes virus proteins and the ER stress markers have been found to co-localize in the alveolar epithelium of patients with IPF (11). In animal models, ER stress in the alveolar epithelium exacerbates lung fibrosis by stimulating alveolar epithelial cell apoptosis (12). STING and ER stress simultaneously contribute to the development of liver fibrosis (13). This study aims to investigate the role of STING and ER stress in AE-IPF and the underlying molecular mechanism.

## Materials and Methods

### Patients

**Figure 1:**
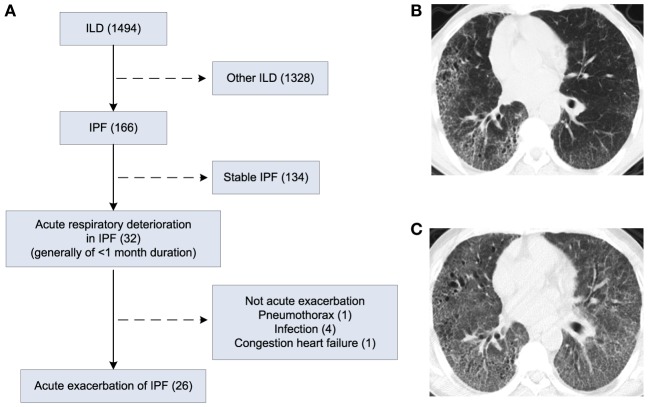
Patient flow chart and chest X-ray and high-resolution computed tomography (HRCT) images of patients. **(A)** Patient flow chart. **(B)** HRCT image of a patient with IPF. **(C)** HRCT image of the patient in D who developed AE-IPF.

This study was approved by the Ethics Committee of Shanghai Pulmonary Hospital (Approval No: 2014FK04, Approved date: February 25, 2014). Of the 1,494 patients, who were admitted to Shanghai Pulmonary Hospital for interstitial lung disease between February 2014 and August 2016, 166 had confirmed IPF according to the 2013 ATS/ERS guidelines (1). Thirty-two patients with IPF developed acute respiratory deterioration within 1 month before hospital admission. Of the 32 cases, 26 met the diagnostic criteria for AE-IPF (1) and were included in the study. The diagnostic criteria for AE-IPF are provided in the Supplementary Material (1). Patient flowchart is displayed in Figure 1A. Written informed consent was obtained from all study participants.

Improvement after treatment was defined as alleviated respiratory symptoms, ≥10% decrease in radiographic lesions, and/or ≥10% improvement in arterial partial pressure of oxygen (PaO_2_) within 1 month of treatment compared to the condition prior to treatment. Deterioration was defined as aggravated respiratory symptoms, continuously expanded radiographic lesions, and/or a progressive decrease in PaO_2_. All study participants with complete data were followed up until August 30, 2016.

### Gene Expression Profiling

Blood samples were collected from study participants before treatment and from healthy individuals who had routine physical examination in the hospital. Total RNA was extracted from the whole blood samples and global gene expression was analyzed. Details are provided in the Supplementary Material.

### Isolation and Analysis of Primary Peripheral Blood Mononuclear Cells (PBMCs)

Primary PBMCs were prepared by density gradient centrifugation of the whole blood samples. The STING ligand, cyclic guanosine monophosphate-adenosine monophosphate (cGAMP, 5 µg/mL, BioVision, Milpitas, CA, USA) was used to stimulate PBMCs at 37°C for 24 h as previously described (14). The primary PBMCs were analyzed by flow cytometry, RT-PCR (15), Western blot (16), and TUNEL assay. Details are provided in the Supplementary Material. PCR primers are displayed as Table S1 in Supplementary Material.

### Cell Culture and Treatment

Mouse macrophage cell line Raw264.7, human lung adenocarcinoma cell line A549, and herpes simplex virus type 1 (HSV-1) were kindly provided by Dr. Qiang Wang (Shanghai Institutes for Biological Sciences, Chinese Academy of Sciences). HSV-1 was propagated and tittered by plaque assay on Vero cells as the previous description (16). Raw264.7 cells and A549 cells were infected with HSV-1. A549 cells were treated with transforming growth factor-β1 (TGF-β1, 5 ng/mL) for 48 h to induce epithelial–mesenchymal transition (EMT), and then infected with HSV-1 (17). Tauroursodeoxycholic acid (TUDCA, 500 µg/mL, Sigma-Aldrich) was added during EMT process (18). Cells were analyzed by Western blot and TUNEL assay. Details are provided in the Supplementary Material.

### Mouse Model of Pulmonary Fibrosis

C57BL/6 male mice were purchased from Shanghai SLAC Laboratory Animal Co., Ltd. The procedures for animal maintenance and experiments were approved by the Institutional Animal Care and Use Committee at Tongji University. Pulmonary fibrosis was induced by intratracheal bleomycin injection (40 µL of 5.0 U/kg, Nippon Kayaku, Japan) as previously described (19). Mice were injected intranasally with 5 × 10^5^ plaque-forming units (pfu) HSV-1 at day 14 after bleomycin injection. Previous study has shown that pulmonary fibrosis is established 14 days after bleomycin injection in mice (20). TUDCA (100 µL of 250 mg/kg) was injected intraperitoneally daily after HSV-1 infection (18). The procedure of mouse experiment is displayed as Figure S1 in Supplementary Material. Details of histology of mouse lung tissue, mouse lung function measurement (21), RT-PCR, Western blot, and ELISA assay for cytokines are described in the Supplementary Material. PCR primers are displayed as Table S1 in Supplementary Material.

### Isolation of Bone Marrow-Derived Macrophages (BMDMs) from Mice

Primary BMDMs were isolated from mice and cultured as previously described (22). Mature mouse BMDMs were identified as the subpopulation with CD11b^+^F4/80^+^ based on flow cytometry (Figure S2 in Supplementary Material).

### Data Analysis

Statistical analyses were performed using GraphPad Prism 5.0 (GraphPad Software, San Diego, CA, USA). Differences between two groups were analyzed using unpaired Student’s *t*-test. Differences among multiple groups were analyzed using one-way analysis of variance and between groups using Bonferroni’s multiple comparison tests. For mouse survival, Kaplan–Meier survival curves were plotted and analyzed by log-rank test. The correlation between STING levels and PaO_2_ was analyzed by Pearson correlation analysis. Results are present as means ± SEM. *p* values ≤0.05 were considered significant.

## Results

### Clinical Characteristics

Mean age, gender distribution, and proportion of cases with a history of cigarette smoking were similar in AE-IPF and stable IPF groups (Table 1). By contrast, significantly higher proportion of patients with AE-IPF (42%) than patients with stable IPF (9%) had a history of recent cold (*p* < 0.01, Table 1). The AE-IPF group also had significantly higher serum mean levels of white blood cell count, C-reactive protein, and lactate dehydrogenase than the stable IPF group (all *p* < 0.01, Table 1), indicating an increased inflammation in the AE-IPF group. Compared with the stable IPF group, the AE-IPF group showed poorer lung function, significantly lower mean PaO_2_, lower mean ratio of arterial oxygen partial pressure and fraction of inspiratory oxygen concentration (*P*/*F*), higher proportion of mechanical ventilation use, and longer hospital stay (all *p* < 0.01, Table 1). The 1-year mortality in the AE-IPF group (69%) was significantly higher than that in the stable IPF group (7%, *p* < 0.01, Table 1), suggesting a poorer prognosis.

**Table 1:** Demographic and clinical characteristic of patients.

	AE-IPF (*n* = 26)	Stable IPF (*n* = 134)	*p*-value
Age, year	65.3 ± 8	66.4 ± 7.8	0.52
Male, gender	26 (100%)	119 (88.8%)	0.16
Smoking	18 (69.2%)	89 (66.4%)	0.15
History of recent cold	11(42.3%)	12 (9%)	0.01*
WBC, ×10^9^/L	11.5 ± 3.2	7.1 ± 2.3	0.01*
CRP, mg/L	31.6 ± 16.1	10 ± 17.6	0.01*
LDH, IU/L	300.3 ± 90.3	216 ± 56.3	0.01*
PH	7.4 ± 0.0	7.4 ± 0.0	0.63
PaO_2_, mmHg	58.4 ± 10.8	75.5 ± 13.6	0.01*
PaCO_2_, mmHg	38.8 ± 7.9	39.2 ± 4.3	0.74
*P*/*F*	140 ± 9.1	269.5 ± 69.1	0.01*
Mechanical ventilation	19 (73.1%)	8 (6%)	0.01*
Length of stay, days	17.8 ± 6.6	6.2 ± 3.5	0.01*
1-year mortality	18 (69.2%)	10 (7.4%)	0.01*

*Data are presented as means ± SEM*.

*p < 0.05.

*CRP, C-reactive protein; LDH, lactate dehydrogenase; P/F, ratio of arterial oxygen partial pressure and fraction of inspiratory oxygen concentration*.

High-resolution computed tomography (HRCT) of a patient with IPF displayed usual interstitial pneumonia (UIP) characterized by bibasal, peripheral predominant reticular opacities with traction bronchiectasis, and honeycombing (Figure 1B). At the onset of AE-IPF, his HRCT demonstrated newly developed diffuse ground glass opacities in both lungs in addition to the existing UIP lesions (Figure 1C).

### STING Signaling Pathway Was Impaired in the PBMCs of Patients with AE-IPF

**Figure 2:**
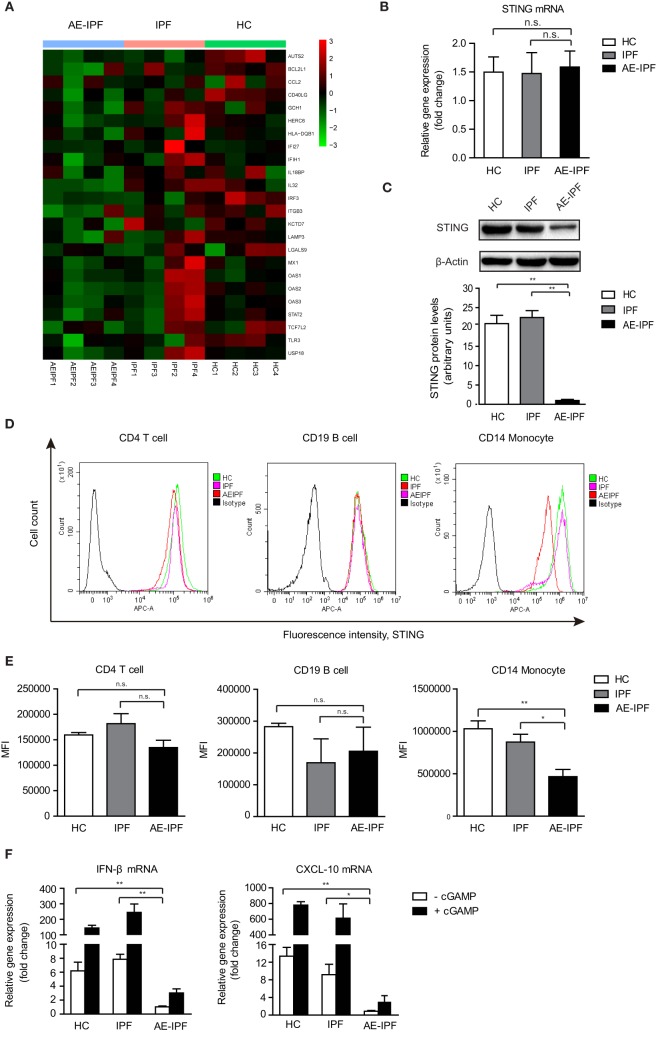
The stimulator of interferon genes (STING) signaling pathway was impaired in the primary PBMCs of patients with AE-IPF. **(A)** Heat map of expression of interferon-regulated genes of 12 study participants from the AE-IPF, IPF, and HC groups (4 study participants of each group). Red color represents increased gene expression levels and green color represents reduced gene expression levels. **(B)** Relative mRNA levels of STING in PBMCs in HC, IPF, and AE-IPF groups (*n* = 12). **(C)** Western blot analysis of STING protein expression in the primary PBMCs of the three groups (*n* = 12). The intensity of the band was normalized to β-actin. **(D)** Flow cytometry histogram of STING expression in CD4 T cells, CD19 B cells, and CD14 monocytes. **(E)** MFI of the flow cytometry assay in human PBMCs were isolated from patients in the IPF and AE-IPF groups and from HCs (*n* = 6). **(F)** Relative mRNA expression of IFNβ and CXCL-10 in PBMCs with (+) or without (−) cGAMP stimulation (*n* = 8). Data are presented as means ± SEM, *p < 0.05; **p < 0.01; n. s., not significant. Abbreviations: AE-IPF, acute exacerbation of idiopathic pulmonary fibrosis; HC, healthy control; PBMC, peripheral blood mononuclear cell; MFI, mean fluorescence intensity; IFNβ, interferonβ.

Stimulator of interferon genes signaling pathway plays a critical role in mediating immune defense against DNA viruses, such as HSV-1 (23). Type I IFNs and inflammatory factors, such as C-X-C motif chemokine 10 (CXCL-10), are downstream factors of the STING signaling pathway (24). Global gene expression profiling revealed a significant downregulation of IFN-regulated signature genes in PBMCs of four patients with AE-IPF (Figure 2A; Table S2 in Supplementary Material) compared with healthy controls (HCs) and patients with stable IPF, suggesting that the STING signaling pathway may be impaired in AE-IPF. Although STING mRNA levels in the primary PBMCs were similar in the AE-IPF, stable IPF, and HC groups (Figure 2B), the STING protein levels in the AE-IPF group were reduced approximately twenty-fold compared with those in the IPF and HC groups (Figure 2C). Flow cytometry further confirmed that STING protein expression was particularly reduced in monocytes but remained unchanged in T and B lymphocytes (Figures 2D,E) in the AE-IPF group.

The mRNA levels of IFNβ and CXCL-10 were significantly downregulated in the PBMCs of the AE-IPF group compared with the IPF and HC groups (Figure 2F). cGAMP stimulated the expression of downstream factors of STING at substantially lower extent in the PBMCs of the AE-IPF group. cGAMP increased the gene expressions of IFNβ and CXCL-10 by 2 times in the AE-IPF group, whereas by more than 200 times in the HC and IPF groups (Figure 2F). Similarly, both baseline and cGAMP-induced mRNA levels of IFNα in PBMCs were lower in the AE-IPF group than in the IPF and HC groups (Figure S3 in Supplementary Material). IFNβ proteins were not detected in the serum of the AE-IPF group but were found in the serum of the HC and IPF groups (Figure S4 in Supplementary Material). These results indicate that patients with AE-IPF may have decreased STING proteins and impaired STING signaling pathway in PBMCs, which may compromise their innate immune function. The impairment of the STING signaling pathway in PBMCs of AE-IPF may be associated with severe viral infection-induced ER stress and the consequent ER functional disorder.

### ER Stress, UPR, and Apoptosis Were Increased in the PBMCs of AE-IPF

**Figure 3:**
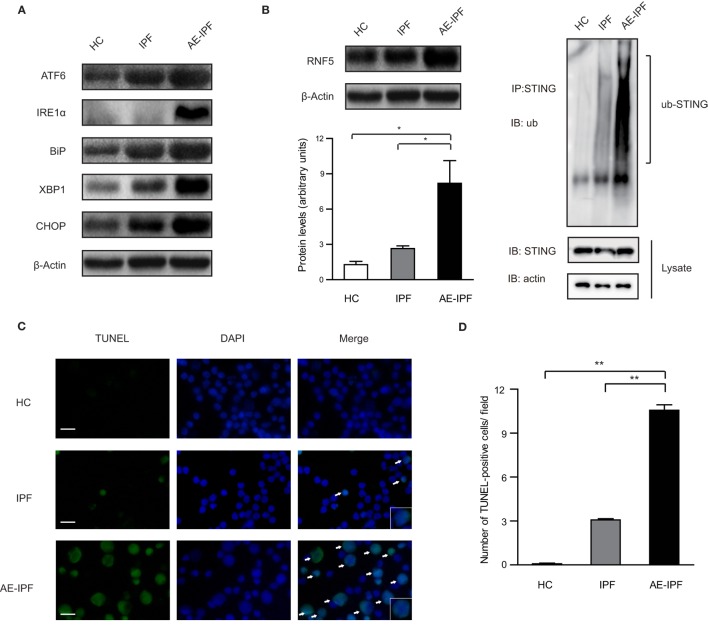
Endoplasmic reticulum (ER) stress and unfolded protein response were activated in the primary peripheral blood mononuclear cells (PBMCs) of patients with acute exacerbation of idiopathic pulmonary fibrosis (AE-IPF). **(A)** Representative Western blot image for the expression of the ER stress markers, activating transcription factor 6 (ATF6), inositol-requiring enzyme 1α (IRE1α), binding immunoglobulin protein (BiP), X-box protein 1 (XBP1), and C/EBP homologous protein (CHOP) in PBMCs from the three groups. β-actin was the loading control. **(B)** Western blot analysis of RNF5 protein expression in the primary PBMCs of the three groups (*n* = 12). The intensity of the band was normalized to β-actin (left). The ubiquitination of endogenous stimulator of interferon genes (STING) in PBMCs from the three groups (right). **(C)** Representative immunofluorescence staining of TUNEL assay. Green signals represent TUNEL positive nucleus and blue signals represent nuclei. White arrows are pointing to apoptotic cells. Magnification: ×400; scale bars represent 20 µm. **(D)** Quantification of the TUNEL assay (*n* = 6). Data are presented as means ± SEM, *p < 0.05; **p < 0.01.

In mammalian cells, UPR is orchestrated by three ER transmembrane proteins: inositol-requiring enzyme 1α (IRE1α), pancreatic endoplasmic reticulum kinase (PERK), and activating transcription factor 6 (ATF6) (9). Western blot (the same samples used in STING protein Western blot) showed that the ER stress markers, ATF6, IRE1α, binding immunoglobulin protein (also known as GRP78), X-box protein 1 (XBP1, the downstream molecular of the IRE1α pathway), and C/EBP homologous protein (CHOP) were upregulated significantly in the PBMCs from the AE-IPF group compared to the IPF and HC groups (all *p* < 0.05, Figure 3A; Figure S5 in Supplementary Material), indicating the activation of ER stress and UPR in the PBMCs of the AE-IPF group. The protein levels of ATF4, a downstream transcription factor of PERK, were not significantly different in the three groups (Figure S6 in Supplementary Material).

E3 ubiquitin ligase ringer protein 5 (RNF5) can catalyze the K48-linked poly-ubiquitination of STING and promote proteasome-dependent degradation of STING (25). In the PBMCs of the AE-IPF group, RNF5 protein levels were increased 8 times of those in the HC group and 2.5 times of those in the IPF group. The ubiquitination was significantly increased in the AE-IPF group compared to the stable IPF and HC groups (Figure 3B), which may cause the STING protein reduction in the PBMCs of AE-IPF. In addition, TUNEL assay showed that PBMC apoptosis was significantly elevated in the AE-IPF group compared with that in the IPF and HC groups (Figures 3C,D). Based on these results, we speculated that ER stress might contribute to the impaired STING signaling pathway in the PBMCs of AE-IPF.

### HSV-1 Infection Triggered ER Stress and Downregulated STING Protein Expression in Raw264.7 and A549 Cells

**Figure 4:**
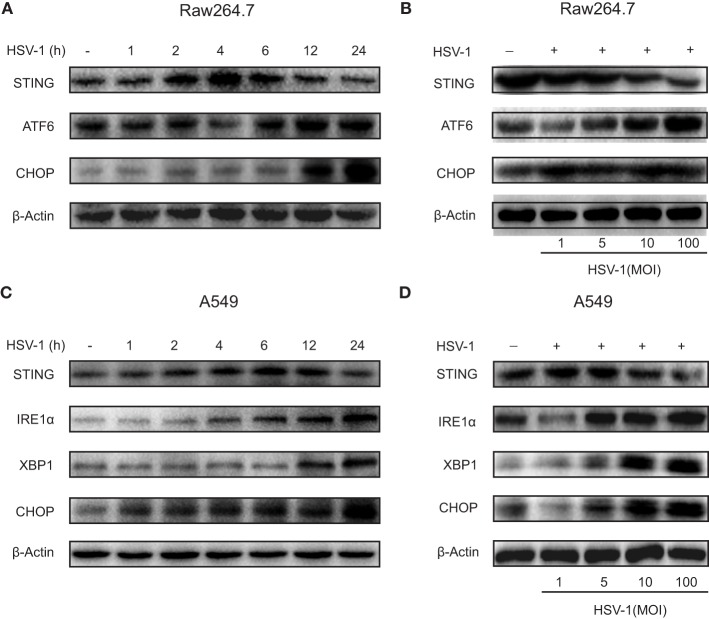
Herpes simplex virus type 1 (HSV-1) infection-induced endoplasmic reticulum stress and downregulated stimulator of interferon genes (STING) protein expression in Raw264.7 cells and A549 cells. **(A)** Representative Western blot image of the time course of the protein expression of STING, activating transcription factor 6 (ATF6), and C/EBP homologous protein (CHOP) in Raw264.7 cells treated with HSV-1 [multiplicity of infection (MOI) 5] for 0, 1, 2, 4, 6, 12, or 24 h. **(B)** Representative Western blot image of the dose response of the protein expression of STING, ATF6, and CHOP in Raw264.7 cells treated with HSV-1 (MOI 1, 5, 10, or 100) for 6 h. **(C)** Representative Western blot image of the time course of the protein expression of STING, inositol-requiring enzyme 1α (IRE1α), X-box protein 1 (XBP1), and CHOP in A549 cells treated with HSV-1 (MOI 5) for 0, 1, 2, 4, 6, 12, or 24 h. **(D)** Representative Western blot image of the dose response of the protein expression of STING, IRE1α, XBP1, and CHOP in A549 cells treated with HSV-1 (MOI 1, 5, 10, or 100) for 6 h.

Stimulator of interferon genes protein expression increased gradually and reached a maximal level 4–6 h after HSV-1 infection, and then gradually reduced to the baseline level 24 h after the infection in both Raw264.7 cells (Figure 4A) and A549 cells (Figure 4C). STING expression reduced progressively as HSV-1 dose increased in both Raw264.7 (Figure 4B) and A549 cells (Figure 4D). ATF6 in Raw264.7 cells and IRE1α in A549 cells were stimulated by HSV-1 infection in a time-dependent (Figures 4A,C) and dose-dependent (Figures 4B,D) manner. CHOP, a proapoptotic protein, was upregulated in both cell lines by HSV-1 infection time dependently (Figures 4A,C) and dose dependently (Figures 4B,D). The two UPR factors, IRE1α and XBP1, were not induced by HSV-1 infection in Raw264.7 cells, neither was ATF6 in A549 cells (Figure S7 in Supplementary Material).

**Figure 5:**
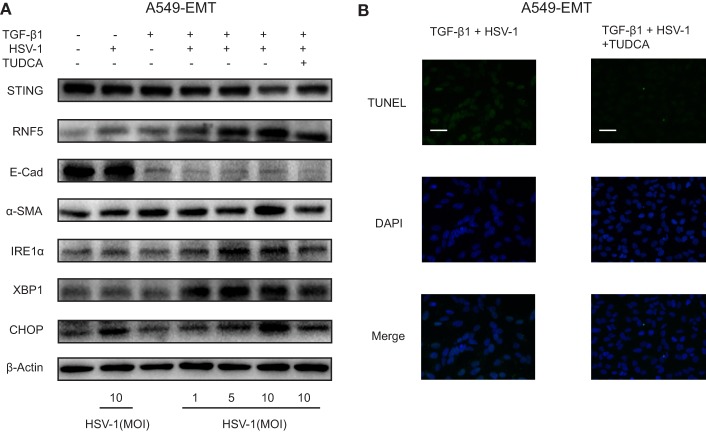
Herpes simplex virus type 1 (HSV-1) infection induced endoplasmic reticulum stress and downregulated stimulator of interferon genes (STING) protein expression in A549-EMT cells. **(A)** Representative Western blot image of the protein expression of STING, RNF5, E-Cad, α-SMA, inositol-requiring enzyme 1α (IRE1α), X-box protein 1 (XBP1), and C/EBP homologous protein (CHOP) in A549 cells treated with herpes simplex virus type 1 (HSV-1) (MOI 0, 1, 5, or 10) for 6 h and transforming growth factor-β1 (TGF-β1) (5 ng/mL) for 48 h to induce epithelial–mesenchymal transition (EMT). A549 cells were treated with tauroursodeoxycholic acid (TUDCA) (500 µg/mL) prior to HSV-1 infection. Western blot was repeated for three times and the three experiments showed similar results. **(B)** Representative immunofluorescence staining for TUNEL assay of A549 cells treated with TGF-β1 and HSV-1. Magnification: ×400; scale bars represent 20 µm. Abbreviation: E-cad, E-cadherin.

The hallmark of IPF is aberrant myofibroblast proliferation. EMT has been considered as a principal source of myofibroblast (26). We treated A549 cells with TGF-β1 to induce EMT and mimic myofibroblast differentiation (A549-EMT cells). The expression of α-SMA, a biomarker for myofibroblast differentiation, was increased substantially by TGF-β1, and the expression of E-cadherin, an epithelial adhesion molecule, was markedly downregulated by TGF-β1 (Figure 5A). STING protein expression was reduced substantially by HSV-1 infection (MOI 10) in A549-EMT cells, whereas was not affected by the same dose of HSV-1 in TGF-β1-untreated A549 cells (Figure 5A). The protein expressions of RNF5, IRE1α, XBP1, and CHOP were stimulated in A549-EMT cells by HSV-1 in a dose-dependent manner (Figure 5A). In addition, TUDCA, which is a small molecule chaperone regulating proper protein folding in ER and can alleviate ER stress, partially reversed the HSV-1-mediated effects. TUDCA treatment increased STING expression and reduced RNF5, IRE1α, XBP1, and CHOP expressions in HSV-1-treated A549-EMT cells (Figure 5A). TUDCA also dramatically reduced the apoptosis of HSV-1-treated A549-EMT cells (Figure 5B). These data suggest that HSV-1 infection may induce ER stress, which consequently reduce STING protein expression by degradation and apoptosis.

### HSV-1 Infection Exacerbated Bleomycin-Induced Pulmonary Fibrosis in Mice and TUDCA Partially Attenuated the Adverse Effects

**Figure 6:**
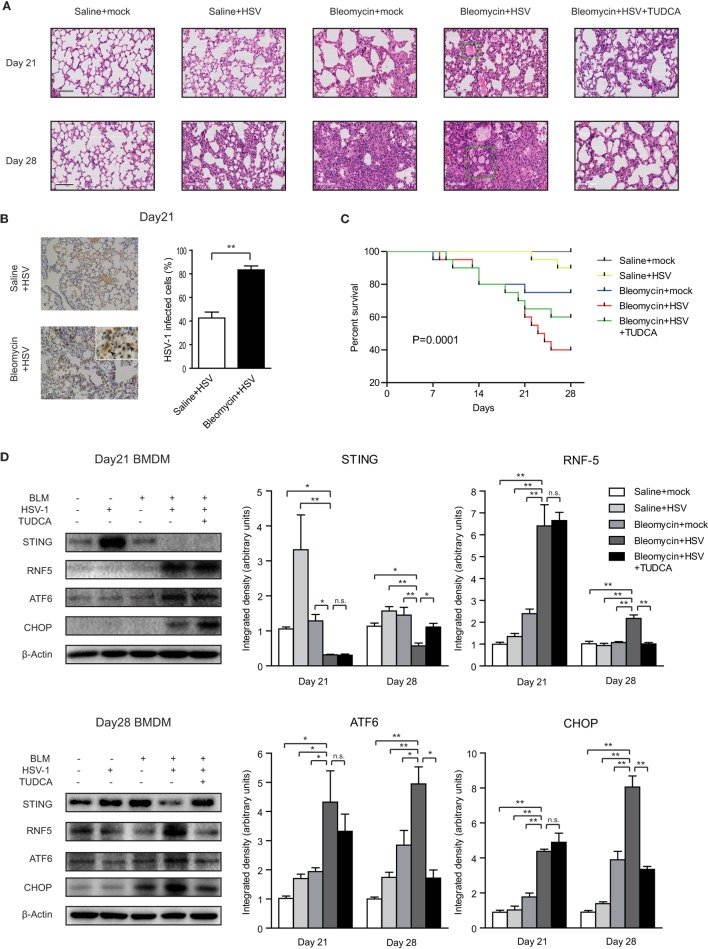
Herpes simplex virus type 1 (HSV-1) infection exacerbated lung injury, induced endoplasmic reticulum stress, and downregulated stimulator of interferon genes (STING) protein expression in mice with bleomycin-induced lung fibrosis. **(A)** Representative image of mouse lung sections with hematoxylin and eosin staining. The microscopic appearance of diffuse alveolar damage (DAD) with hyaline membranes (box) was founded in the lungs from mice treated with Bleomycin and HSV-1. Original magnification: ×200. Scale bars represent 100 µm (*n* = 10). **(B)** Immunohistochemistry analysis of HSV-1 particle deposition in mouse lung tissues (*n* = 10). Solid black arrows are pointing to cells infected by HSV-1. Percentages of cells infected by HSV-1 were quantified. Original magnification: ×200, scale bars represent 100 µm. **(C)** Kaplan–Meier analysis of mouse survival (*n* = 20). **(D)** The expression of STING, RNF5, activating transcription factor 6 (ATF6), and CHOP in primary bone marrow-derived macrophages (BMDMs) isolated from mice on day 21 and day 28 after bleomycin injection. Left: representative western blot image. Right: densitometry analysis of the western blot bands (*n* = 5). The intensity of the protein bands was normalized to β-actin. Data are presented as means ± SEM, *p < 0.05; **p < 0.01; n.s., not significant.

Histological examination showed that HSV-1 infection exacerbated the inflammation and collagen deposition in the lung of mice with bleomycin-induced pulmonary fibrosis (Figure 6A). The diffuse alveolar damages in the mice with pulmonary fibrosis, which were characterized by interstitial edema, intra-alveolar hemorrhage, alveolar epithelial denudation, and hyaline membranes, resembled the lung histopathology of patients with AE-IPF (Figure 6A; Figure S8A in Supplementary Material). HSV-1 infection increased the acute lung injury score and fibrosis score in mice with bleomycin-induced pulmonary fibrosis (Figure S8B in Supplementary Material). The accumulation of HSV-1 virus particles in lung tissues was significantly higher in the mice receiving both bleomycin and HSV-1 than in the mice treated with HSV-1 alone (Figure 6B). HSV-1 infection also reduced forced vital volume (FVC) and lung compliance in the mice with bleomycin-induced lung fibrosis (Figure S8C in Supplementary Material). The mortality of mice treated with bleomycin and HSV-1was the highest, and approximately 75% of the mice died within 14 days after HSV-1 infection (Figure 6C).

Primary BMDMs were isolated from mice on day 21 or day 28 after bleomycin injection. STING protein expression was dramatically increased in the BMDMs of mice without bleomycin-induced lung fibrosis after HSV-1 infection (Figure 6D). By contrast, STING protein levels were entirely absent in the BMDMs isolated on day 21 from the mice treated with bleomycin and HSV-1 and substantially reduced in the BMDMs isolated on day 28 (Figure 6D). Contrarily, RNF5, ATF6, and CHOP protein levels were increased in BMDMs from the mice treated with bleomycin and HSV-1 (Figure 6D). In mouse lung tissues, although STING protein expression was not changed, ATF6 and CHOP protein expressions were increased considerably by HSV-1 infection (Figure S9 in Supplementary Material). The ratio of phosphorylated IFN regulatory factor 3 (IRF3) to total IRF3 in the lung tissue of the mice treated with bleomycin and HSV-1 was increased by three times compared with that of the mice treated with saline and mock infection on day 7 after HSV-1 infection (day 21 after bleomycin injection) but remained unchanged on day 14 after HSV-1 infection (day 28 after bleomycin injection) (Figure S9 in Supplementary Material). In addition to inducing ER stress in the lung and BMDMs, HSV-1 infection also increased protein levels in BALF, promoted inflammatory cytokine release, including interleukin-6 (IL-6), tumor necrosis factor-α (TNF-α), and monocyte chemoattractant protein-1 (MCP-1), and reduced anti-inflammation cytokine IL-10 production (Figures S10A–C in Supplementary Material).

Notably, TUDCA, an inhibitor of ER stress, exerted protective effects in the mice treated with bleomycin and HSV-1. TUDCA reduced bleomycin-induced pneumonitis and fibrosis and improved lung function and survival (Figures 6A,C; Figures S8A–C in Supplementary Material). Consistent with the results from Raw264.7 and A549 cells, TUDCA also partially restored STING protein expression and reduced RNF5, ATF6, and CHOP levels in mouse BMDMs isolated on day 28, even though STING protein was nearly absent on day 21 (Figure 6D). In lung tissues, TUDCA reduced HSV-1 infection-mediated upregulation of ATF6 and CHOP (Figure S9 in Supplementary Material). Furthermore, TUDCA decreased the content of total protein in BALF and IL-6, TNF-α, and MCP-1 expression, and increased IL-10 levels (Figures S10A–C in Supplementary Material). Taken together, these data indicate that HSV-1 infection may stimulate ER stress, which may lead to STING deficiency by stimulating RNF5 pathway, ultimately causing an exacerbation of bleomycin-induced lung injury in mice.

### STING Protein Levels in PBMCs Reflected the Effectiveness of Therapies for AE-IPF

**Figure 7:**
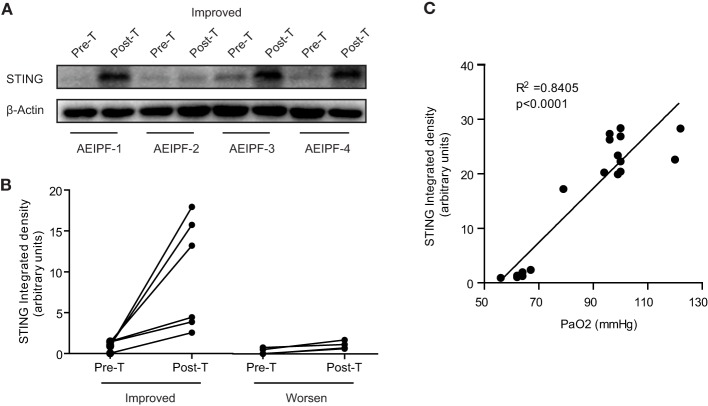
Stimulator of interferon genes (STING) protein levels in peripheral blood mononuclear cells (PBMCs) reflected the prognosis of acute exacerbation of idiopathic pulmonary fibrosis (AE-IPF). **(A)** Representative western blot image of STING protein expression in PBMCs isolated from 10 patients with AE-IPF (6 cases of improvement + 4 cases of deterioration) before and after the treatment. **(B)** Quantification of the Western blot in panel **(A)**. Six patients with improvement showed increased STING expression in PBMCs after treatment. Four patients with deterioration showed unchanged STING expression after treatment. **(C)** STING protein expression levels positively and significantly correlated with pre-treatment PaO_2_ levels. Six cases were randomly selected from healthy control, stable IPF, and AE-IPF groups, respectively. Abbreviations: pre-T, pre-treatment; post-T, post-treatment.

To investigate the dynamic changes of STING at different disease status, we collected primary PBMCs from 10 patients with AE-IPF before and after treatment. Of the 10 patients, 6 showed improvement and 4 had deterioration after treatment. Before treatment, STING protein levels in all the 10 patients were very low, whereas after treatment, STING protein levels were substantially increased in patients showing improvement and remained at low levels in patients showing deterioration (Figures 7A,B). STING protein levels may indicate the effectiveness of therapies for AE-IPF. Furthermore, Pearson correlation analysis revealed a significant positive correlation between pre-treatment STING protein levels and PaO_2_ level (*R*^2^ = 0.8405, *p* < 0.0001, Figure 7C). Thus, STING protein levels in PBMCs may reflect the severity of pulmonary dysfunction in IPF.

## Discussion

The current study showed 15.7% patients with IPF (26/166) experienced at least one episode of acute exacerbation during a 30-month follow-up period and the 1-year mortality rate of AE-IPF was 69%. These findings are consistent with the previous reports, which have found that the annual incidence of AE-IPF is 5–15% and the short-term mortality of AE-IPF is higher than 50% (27–29). Viral infection may be the “second hit” to further exacerbate genetic or environmental factor-mediated lung injuries (3, 20, 30, 31). In the current study, 42.3% of patients with AE-IPF had a history of cold before the onset of AE-IPF and HSV-1 infection exacerbated bleomycin-induced lung fibrosis in mice.

**Figure 8:**
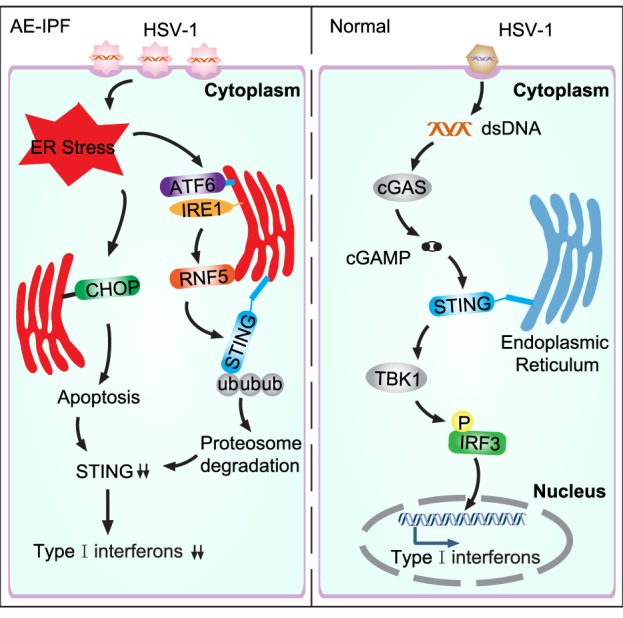
Schematic diagram of virus-induced endoplasmic reticulum (ER) stress and the consequent downregulation of the stimulator of interferon genes (STING) pathway in acute exacerbation of idiopathic pulmonary fibrosis (AE-IPF). In normal condition, herpes simplex virus type 1 (HSV-1) infection activates the cytosolic DNA sensor, cyclic GMP-AMP synthase (cGAS), which then generates cGAMP. cGAMP binds STING and activates the downstream signaling pathways. TANK-binding kinase 1 (TBK1) is subsequently recruited to phosphorylate IFN regulatory factor 3 (IRF3), leading to type I interferons expression. In AE-IPF, severe viral infection activates ER stress and unfolded protein response (UPR), which then promotes STING degradation *via* stimulating RNF5. ER stress induces apoptosis, which further decreases STING production and downregulates type I interferons.

Stimulator of interferon genes plays a key role in activating host antiviral responses. In normal physiological condition, HSV-1 infects cells and the viral DNA enters the cell, which triggers the cytosolic DNA sensor, cyclic GMP-AMP synthase, to produce the second messenger, 2′3′-cGAMP. The 2′3′-cGAMP binds STING to activate TANK-binding kinase 1, resulting in IRF3 phosphorylation, which consequently induces the expression of type I IFNs and innate immune response (Figure 8) (23, 29). Infection of STING-deficient mice failed to induce IFN-β and pro-inflammatory cytokine secretion (32). Furthermore, STING-deficient mice seemed highly unlikely to survive HSV-1 infection (6, 33). Consistent with these findings, our data show that STING deficiency may contribute to viral infection-mediated AE-IPF. Severe viral infection may trigger UPR and ER stress, inducing RNF5 over-expression so to promote STING degradation *via* ubiquitination (Figure 8). The activated UPR can also trigger apoptosis, which further reduce STING production (Figure 8). STING deficiency may then reduce IFN expression and compromise host immune response, ultimately leading to the onset of AE-IPF. Our findings appear to support this AE-IPF scenario. We found that HSV-1 infection stimulated UPR and ER stress in Raw264.7 cells, A549 cells, and primary mouse BMDMs and lung tissue of mice treated with bleomycin and HSV-1. UPR and ER stress were also detected in the primary PBMCs of patients with AE-IPF. STING deficiency was detected in those cells with ER stress, and the expression of the downstream factors of STING, including IFN-regulated gene and cytokines that are involved in immune response such as CXCL-10, was downregulated in the STING-deficient cells. Our results from the TUDCA treatment appear to further support the scenario. TUDCA, a known inhibitor of ER stress, not only partially alleviated HSV-1 induced ER stress and partially restored STING protein levels in A549-EMT cells and the primary BMDMs of mice treated with bleomycin and HSV-1 but also attenuated the apoptosis of HSV-1 infected A549-EMT cells and improved the survival of mice treated with bleomycin and HSV-1.

Liu et al. reported that STING expression was induced at both mRNA and protein levels in A549 cells infected with HSV-1 (MOI of 0.1) (34, 35). Surprisingly, HSV-1 infection with high dose (MOI of 10 or 100) downregulated STING protein expression in our study. In addition to ER stress, other posttranslational regulatory mechanisms may also contribute to the STING deficiency in AE-IPF. For example, the autophagy-related serine/threonine protein kinases ULK1 and ULK2 can specifically phosphorylate S366 in STING to promote STING degradation (36). Recently, it was reported that HSV-1 infection induced tripartite motif-containing protein 29 (TRIM29) expressions in human airway epithelial cells. The E3 ubiquitin ligase TRIM29 inhibited the antiviral innate immune response by promoting the STING ubiquitination and degradation (37). Thus, the causal association between ER stress and STING deficiency in AE-IPF needs to be further investigated.

Notably, the positive correlation between pre-treatment STING and PaO_2_ levels suggests that monocyte STING levels appear to reflect the severity of pulmonary dysfunction in IPF and may be an effective biomarker to evaluate AE-IPF. Furthermore, the association between restoration of STING levels and post-treatment improvement indicates STING levels may reflect the effectiveness of therapies for AE-IPF.

In conclusion, severe viral infection could induce ER stress to promote STING degradation by upregulating RNF5 and to reduce STING production by inducing apoptosis of immune cells, resulting in STING deficiency and immune response disorder, which ultimately may trigger AE-IPF.

## Ethics Statement

This study was approved by the Ethics Committee of Shanghai Pulmonary Hospital (approval no: 2014FK04, approved date: February 25, 2014). Written informed consent was obtained from all participants.

## Author Contributions

Experimental design: H-PL, CW, HQ, DW, TC, and LS; data acquisition and analysis: HQ, DW, TC, LS, S-SC, Y-RW, QW, M-MZ, Q-HL, YH, YuanZ, YingZ, Y-LS, FZ, L-QL, N-YZ, S-LL, L-LZ, CW, and H-PL; writing the manuscript: HQ, DW, and H-PL. All authors read and approved the final manuscript.

## Conflict of Interest Statement

The authors declare that the research was conducted in the absence of any commercial or financial relationships that could be construed as a potential conflict of interest.
