# Supplementary material for: Stimulator of Interferon Genes Deficiency in Acute Exacerbation of Idiopathic Pulmonary Fibrosis
Source: Front Immunol. 2017 Dec 11;8:1756. doi: 10.3389/fimmu.2017.01756 (PMC5732537; doi:10.3389/fimmu.2017.01756)
Supplement: Supplementary file 1 [file Presentation_1.PDF]

Online data supplement

CONTENTS

Supplementary Methods

Supplementary Figure Legends

1. Supplementary Figure S1. Mouse modeling workflow
2. Supplementary Figure S2. Flow cytometry analysis of primary mouse bone-marrow-derived macrophages
3. Supplementary Figure S3. IFNα expression in human PBMCs
4. Supplementary Figure S4. Serum IFNβ levels in human samples
5. Supplementary Figure S5. ER stress and UPR were activated in the primary PBMCs of patients with AE-IPF
6. Supplementary Figure S6. ATF4 expression in PBMCs
7. Supplementary Figure S7. The effects of HSV-1 infection on the expression of IRE1α and XBP1 in Raw264.7 cells and ATF6 in A549 cells
8. Supplementary Figure S8. HSV-1 infection exacerbated bleomycin-induced lung fibrosis
9. Supplementary Figure S9. HSV-1 infection induced ER stress and down-regulated STING protein expression in mice with bleomycin-induced lung fibrosis
10. Supplementary Figure S10. HSV-1 infection exacerbated bleomycin-induced lung inflammatory response

Supplementary Table

1. Primers used in quantitative real-time PCR assays.
2. Microarray raw data

Supplementary Methods

1. PATIENTS AND HEALTHY CONTROLS

A total of 30 healthy individuals aged 40-80 years were enrolled from the medical examination center of Shanghai Pulmonary Hospital between February 2014 and January 2016. All patients and healthy controls signed the written informed consent to participate in the study. The criteria for AE-IPF are: (1) confirmed diagnosis of IPF; (2) unexplained worsening or development of dyspnea within 30 days; (3) High resolution computed tomography (HRCT) shows new bilateral ground glass abnormality and/or consolidation superimposed in addition to the existing reticular or honeycomb pattern; (4) exclusion of alternative causes, including left heart failure, pulmonary embolism, or identifiable cause of acute lung injury.

2. EXPERIMENTS ON PATIENTS' SAMPLES

Gene Expression Profiling Using Human Transcriptome Array 2.0

PaxGene tubes (Qiagen, Hombrechtikon, Switzerland) were used to collect whole blood samples from 12 study participants of the following three groups (4 from each group): healthy control (HC), stable IPF (IPF), and AE-IPF groups. Total RNA was extracted from the blood samples using Paxgene Blood RNA Kit (Qiagen, Hombrechtikon, Switzerland) according to the manufacturers' protocol. RNA concentrations and A260 nm/A280nm ratio were determined using a NanoDrop 1000 spectrophotometer (Thermo Scientific, Wilmington, DE, USA). RNA quality was analyzed by the Agilient Bioananlyzer 2100 (Agilent Technologies, Santa Clara, CA, USA). Global gene expression analysis was performed by Shanghai South Gene Technology Company using Affymetrix GeneChip® Human Transcriptome Array 2.0. Gene expression profile of the three groups was compared using Anova analysis, and a list of 2-fold up- or down-regulated genes was generated. Statistical significance was defined as $p < 0.05$. Results for interferon-regulated genes are shown as a heat map, with shades of red denoting up-regulated genes and shades of green denoting down-regulated genes.

qRT-PCR

Peripheral-blood mononuclear cells (PBMCs) from patients and healthy controls were isolated using the Ficoll-Hypaque (Lymphoprep^{TM}) density gradient centrifugation (STEMCELL Technologies, Vancouver, British Columbia, Canada). Total RNA was extracted using the EZgene^{TM} Blood RNA Miniprep Kit (BIOMIGA, San Diego, CA, USA). Reverse transcription was conducted using the ReverTra Ace qPCR RT Kit (TOYOBO, Osaka, Japan). qRT-PCR was performed using Thunderbird SYBR qPCR Mix Kit (TOYOBO, Osaka, Japan) and Taqman Gene Expression Assay (Applied Biosystems). The levels of gene expression were normalized against β-actin gene expression. DNA sequences of PCR primers are displayed in **Supplementary Table S1**.

Western Blot

Cell lysates were prepared using RIPA buffer (Biotechwell, Shanghai, China) supplemented with a protease inhibitor cocktail (Roche, Shanghai, China). Protein concentration was determined using the BCA Protein Assay Kit (Biotechwell, Shanghai, China). For the analysis of STING, ER stress markers and RNF-5 protein expression, 15-20 μg of lysates were resolved in 10-12% SDS-PAGE Tris Bis gel and transferred to nitrocellulose membranes. The membranes were blocked with 5% w/v bovine serum albumin (BSA) in TBST (Sigma-Aldrich), probed with anti-STING Ab

(1:1000, R&D systems), anti-ATF6 Ab (1:1000, Abcam), anti-IRE1α Ab (1:1000, Cell Signaling), anti-BiP Ab (1:1000, Cell Signaling), anti-XBP1 Ab (1:1000, Abcam), anti-CHOP Ab (1:1000, Cell Signaling), anti-ATF4 Ab (1:1000, Cell Signaling), anti-RNF5 Ab (1:1000, Abcam), or anti-β-actin (1:2000, Arigo biolaboratories) followed by the horseradish peroxidase (HRP) labeled Secondary Abs (1:2000, Huabio, Hangzhou, China). The membranes were scanned using the ChemiDoc™ XRS+ System (Bio-Rad). Relative fluorescence intensity of the protein bands was analyzed using Image Lab™ Software (Bio-Rad). Protein levels are expressed as arbitrary fluorescent units and normalized using the housekeeping gene β-action.

Immuno-precipitation

For denaturing immunoprecipitation, cells were lysed in 1% SDS buffer (50mMTris-HCl pH 7.5, 150mMNaCl, 1% SDS, 10mMDTT) and denatured by heat for 30 minutes. The cell lysates were centrifuged and diluted with Lysis buffer (50mMTris-HCl pH 7.5, 150mM NaCl, 1mMEDTA, 1% Triton X-100) until the concentration of SDS was decreased to 0.1%. The diluted lysates were immunoprecipitated with anti-STING antibodies for four hours to overnight at 4˚C before adding protein A/G agarose for two hours and then analyzed by immunoblotting with antiubiquitin.

Stimulation of Peripheral Blood Mononuclear Cells (PBMCs)

PBMCs (1x 10^6) from patients or healthy controls were suspended in 1 mL RPMI1640 containing 10% fetal bovine serum (FBS, Gibco). Subsequently, the cells were treated with cGAMP (5 µg/mL, BioVision, Milpitas, CA, USA) for 24 hours. Total RNA was extracted and qRT-PCR was performed to analyze gene expression.

Flow Cytometry of PBMCs

PBMCs (2x10^6) from patients or healthy controls were stained with cell surface markers (CD4-FITC, CD19-PE, and CD14-FITC, BD Biosciences). Cells were fixed with paraformaldehyde, permeabilized, and subsequently stained with anti-STING Ab (1:1000, R&D systems) to detect STING. Intracellular antigen STING was detected by Alexa Fluor® 647 Goat anti-mouse IgG antibody (5 µl per million cells, BioLegend). Flow cytometry analysis was performed using CytoFLEX Flow Cytometer system (Backman Coulter). Mean fluorescence intensity (MFI) was analyzed using CytExpert experiment-based software (Backman Coulter).

Enzyme-linked Immunosorbent Assay (ELISA)

The serum levels of IFNβ in AE-IPF patients were measured by ELISA (PBL assay science, Piscataway, USA) according to the manufacturer’s protocols.

TUNEL assay

PBMCs from patients and healthy controls were isolated by Lymphoprep™ density gradient centrifugation, re-suspended in RPMI1640 containing 10% FBS and cultured at 37°C for 6 hours. Apoptosis was evaluated using In Situ Cell Death Detection Kit (Roche) based on the manufactures’ standard procedures. Images of the cells were collected using Leica laser scan confocal microscopy. The average number of apoptotic cells in five random visual fields per section was used for statistical analysis.

3. EXPERIMENTS ON RAW264.7 CELLS AND A549 CELLS

Cell Culture

Mouse macrophage-like cell line Raw264.7 and the human lung adenocarcinoma cell line A549 were cultured in DMEM containing 10% FBS, supplemented with 1% penicillin-streptomycin (Invitrogen).

Herpes Simplex Virus type 1

Herpes simplex virus type 1 (HSV-1) was kindly provided by Dr. Qiang Wang (Shanghai Institutes for Biological Sciences, Chinese Academy of Sciences). HSV-1 was propagated and tittered by plaque assays on Vero Cells as previously described.

Epithelial-Mesenchymal Transition (EMT) Induction and TUDCA treatment

A549 cells were incubated in 6-well plates and treated with or without TGF-β1 (5 ng/mL) for 48h. At 70%-80% confluency, the cells were washed three times with DMEM without serum and then cultured in DMEM containing 2% FBS. The cells were then treated with Tauroursodeoxycholic acid (TUDCA, 500 µg/mL, Sigma-Aldrich) for 12 hours. Subsequently, the A549 cells were infected with HSV-1 for 6 hours. Media were removed, and whole cell protein lysates were prepared. The protein lysates were used for Western blot analysis. A549 cell apoptosis was analyzed by TUNEL assay.

4. MOUSE MODEL

Mouse Model of Pulmonary Fibrosis

C57BL/6 male mice aged 6-8 weeks were purchased from Shanghai SLAC Laboratory Animal CO., Ltd. All animal procedures were approved by the Institutional Animal Care and Use Committee at Tongji University. On day 0, all mice were anesthetized by intraperitoneal injection of sodium pentobarbital (100 µL of 150 mg/kg body weight solution in saline, Bio-Light Biotech, Shanghai, China). Mice were injected intratracheally with bleomycin (40 µL of 5.0U/kg body weight solution in saline, Nippon Kayaku, Japan) to induce lung fibrosis. Mice in the saline + mock group and saline + HSV group received the same volume of saline by intratracheal injection.

Viral Infection and TUDCA Treatment

On day 14 after bleomycin injection, all mice were anesthetized by intraperitoneal injection of sodium pentobarbital (100 µL of 200 mg/kg body weight solution in saline). The mice were inoculated intranasally with $5x10^5$ plaque forming units (pfu) HSV-1 diluted in 20 µL of sterilized PBS. Mice with mock infection were inoculated intranasally with 20 µL of sterilized PBS. Mice were injected intraperitoneally with TUDCA (100 µL of 250 mg/kg body weight solution in saline) daily after HSV-1 infection. Controls for TUDCA-treated group were injected with 100 µL of saline (**Supplementary Figure S1**).

Histological Analysis

Mice were sacrificed on day 21 or day 28. Mouse lung tissues were fixed in 4% paraformaldehyde, embedded in paraffin, sectioned, and mounted on adhesion microscope slides. Tissue sections were stained with hematoxylin and eosin or Masson's trichrome. Histologic slides for inflammation and fibrosis were scored by two experienced pathologists. The score standards were determined by the Ashcroft score on lung histology. For the detection of HSV-1 infection, lung tissue sections were stained using anti-HSV-1 antibody (1:200, Abcam) according to previously

described.

Mouse Bone-Marrow-Derived Macrophage (BMDM) Isolation
Primary mouse BMDMs were isolated from bone marrow, which was collected from the femurs and tibia of mice. The BMDMs were re-suspended in RPMI1640 supplemented with 10% FBS, 50 ng/mL granulocyte-macrophage colony stimulating factor (GM-CSF, PEPROTECH), and 10 nM L-glutamine (Gibco). The culture media were changed every 3 days. On day 7, the BMDMs were harvested for further experiment. Mature BMDMs were defined as CD11b+F4/80+ subpopulations by flow cytometry (Figure S2 in the online supplement).

Pulmonary Function Measurement
Mice were anesthetized by intraperitoneal injection of sodium pentobarbital. Anesthetized mice underwent tracheostomies followed by intubation of 18G intravenous catheter. Mice were placed in a body plethysmograph of the Pulmonary Maneuvers System (DSI's Buxco Electronics). All procedures were performed according to the manufacturer's protocols and previously described. In every animal, three acceptable measurements were recorded, and the average was then calculated. We focused on forced vital volume (FVC), forced expiratory volume in 50millisecond (FEV50), and dynamic compliance (Cdyn).

Lung Collagen Measurements
Lung tissues (30-40 mg) were used to measure the content of hydroxyproline by the alkaline hydrolysis method based on the manufacturer's instructions (Nanjing Jiancheng Bioengineering Institute). The absorbance was measured at 550 nm (Epoch2 microplate reader, BioTek). Hydroxyproline content in lung tissues was calculated using the standard formula given in the instructions.

Cytokine Analysis
For the determination of cytokine interleukin-6 (IL-6), tumor necrosis factor-α (TNF-α), interleukin-10 (IL-10), and monocyte chemoattractant protein-1 (MCP-1) mRNA in lung homogenates, total RNA was extracted from 10-15 µg lung tissue homogenate using the EZgene™ Tissue RNA Miniprep Kit (BIOMIGA, San Diego, CA, USA). The RNA (1 µg) was reverse transcribed into cDNA using random primers and RT Enzyme Mix (TOYOBO, Osaka, Japan). The cDNA was amplified by PCR using primers for murine IL-6, TNF-α, IL-10, MCP-1, and GAPDH. Cycling parameters used for qPCR were: pre-denaturation 95°C, 60 seconds; denaturation 95°C, 15 seconds; annealing/extension 60°C, 60 seconds for 40 cycles. After normalizing data to GAPDH, expression relative to healthy control mice was calculated by the ΔΔCt method. The levels of cytokine IL-6, TNF-α, IL-10, and MCP-1 proteins in lung homogenates were measured by ELISA (Neobioscience technology, Shenzhen, China) according to the manufacturer's protocols.

STING/IRF3 Signaling Pathway Activation in lung tissues
To evaluate the activation of STING/IRF-3 pathway, Western bolt analysis of STING and IRF3 phosphorylation in lung homogenates were performed. Anti-pIRF3 and anti-IRF3 (1:1000, Cell signaling) were used.

Supplementary Figure and Figure Legends

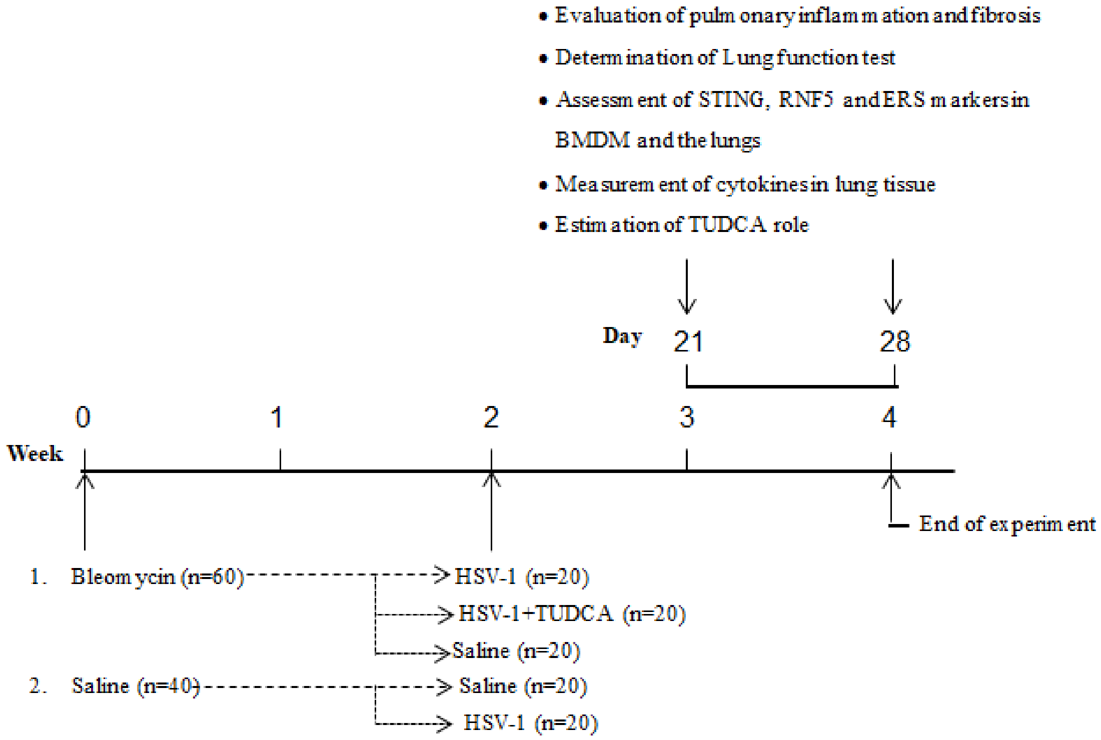

Supplementary Figure S1 Mouse modeling workflow

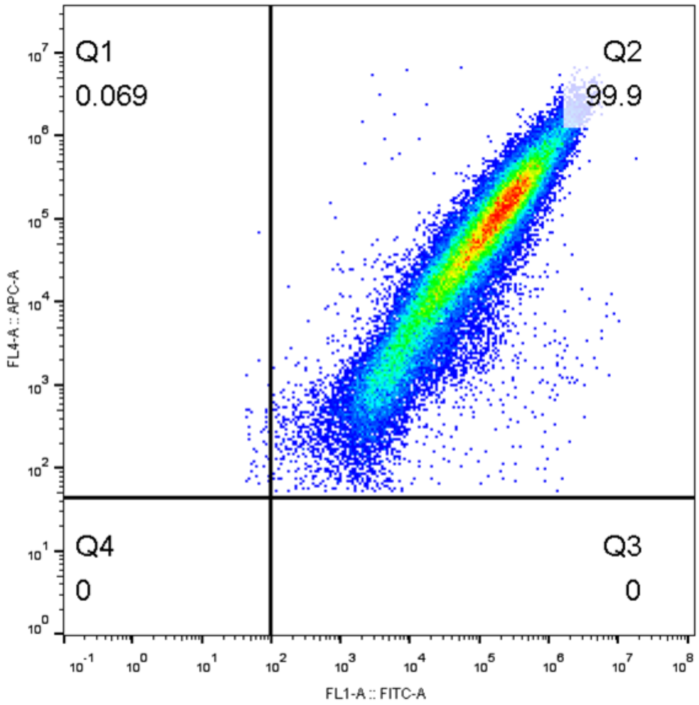

Supplementary Figure S2 Flow cytometry analysis of primary mouse bone-marrow-derived macrophages (BMDM)

Primary mouse mature BMDMs were defined as CD11b and F4/80 double positive cells by flow cytometry analysi**s.**

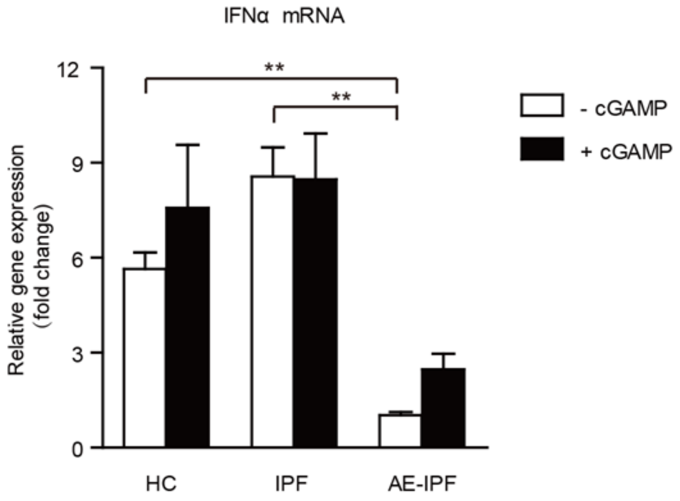

Supplementary Figure S3. IFNα expression in human PBMCs

IFNα mRNA levels in PBMCs from patients and healthy controls with (+) or without (-) cGAMP stimulation (n=8). Data are presented as means ± SEM, ** *p* <0.01. Abbreviation: IFNα: interferon α;

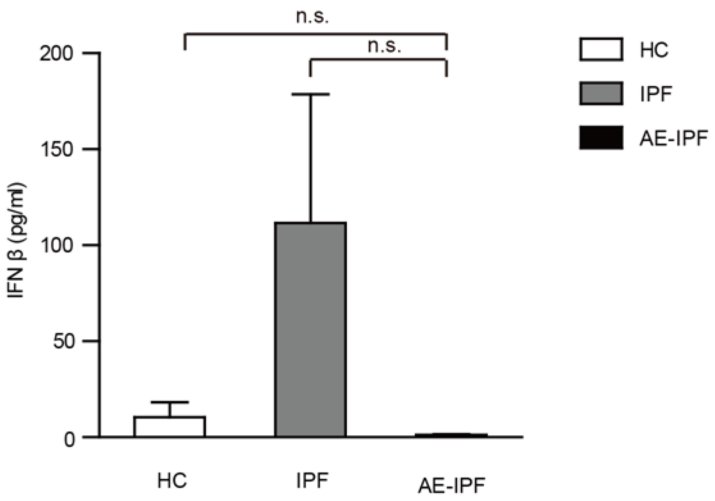

Supplementary Figure S4. Serum IFNβ levels in human samples
IFNβ levels in human serum from patients and healthy controls (n=10). Data are presented as means ± SEM, n. s., not significant.

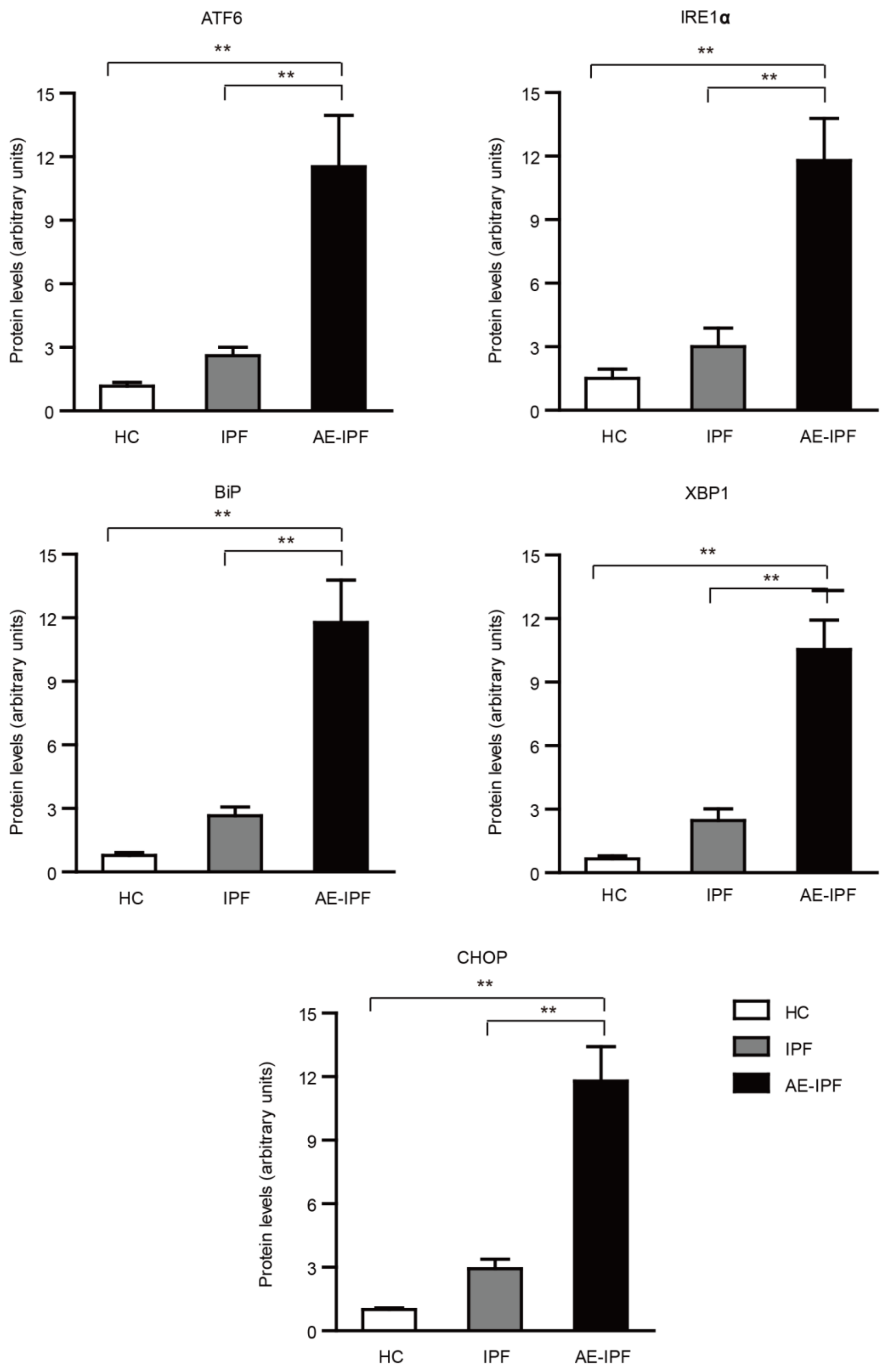

Supplementary Figure S5. ER stress and UPR were activated in the primary PBMCs of patients with AE-IPF

Western blot analysis of ATF6, IRE1α, BiP, XBP1, and CHOP protein expression in PBMCs from patients and healthy controls. Densitometry analysis of the Western blot bands (n=12). The intensity of the protein bands was normalized to β-actin. Data are presented as means ± SEM, * p <0.05; ** p <0.01.

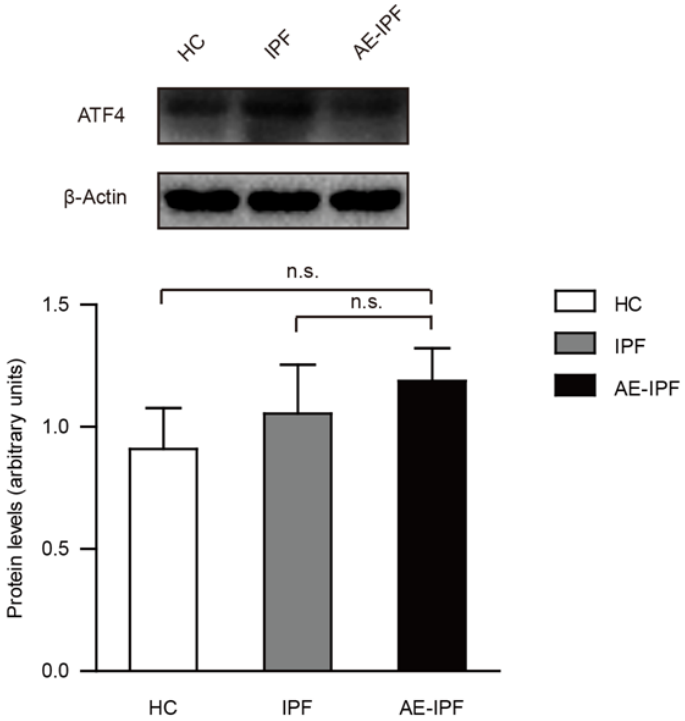

Supplementary Figure S6. ATF4 expression in human PBMCs
Western blot analysis of ATF4 protein expression in PBMCs from patients and healthy controls. Top: representative western blot bands of ATF4 and β-actin. Bottom: quantification of Western blot bands. The intensity of the band was normalized to β-actin (n=12). Abbreviation: ATF4: activating transcription factor 4. Data are presented as means ± SEM, n. s., not significant.

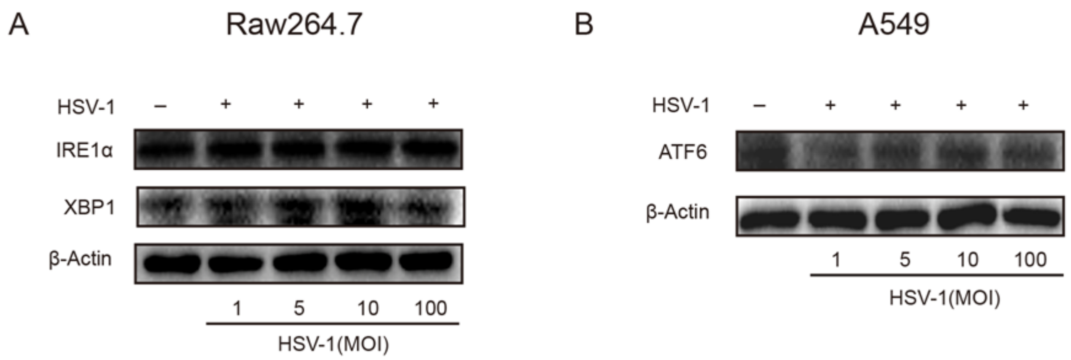

Supplementary Figure S7 The effects of HSV-1 infection on the expression of IRE1α and XBP1 in Raw264.7 cells and ATF6 in A549 cells

A. Representative Western blot image showing the expression of IRE1α and XBP-1 in Raw264.7 cells with or without HSV-1 infection (MOI 1, 5, 10, or 100). **B**. Representative Western blot image showing the expression of ATF6 protein in A549 cells with or without HSV-1 infection (MOI 1, 5, 10, or 100). The experiments were repeated three times. Abbreviation: IRE1α: inositol-requiring enzyme 1α; XBP1: X-box protein 1; ATF6: activating transcription factor 6.

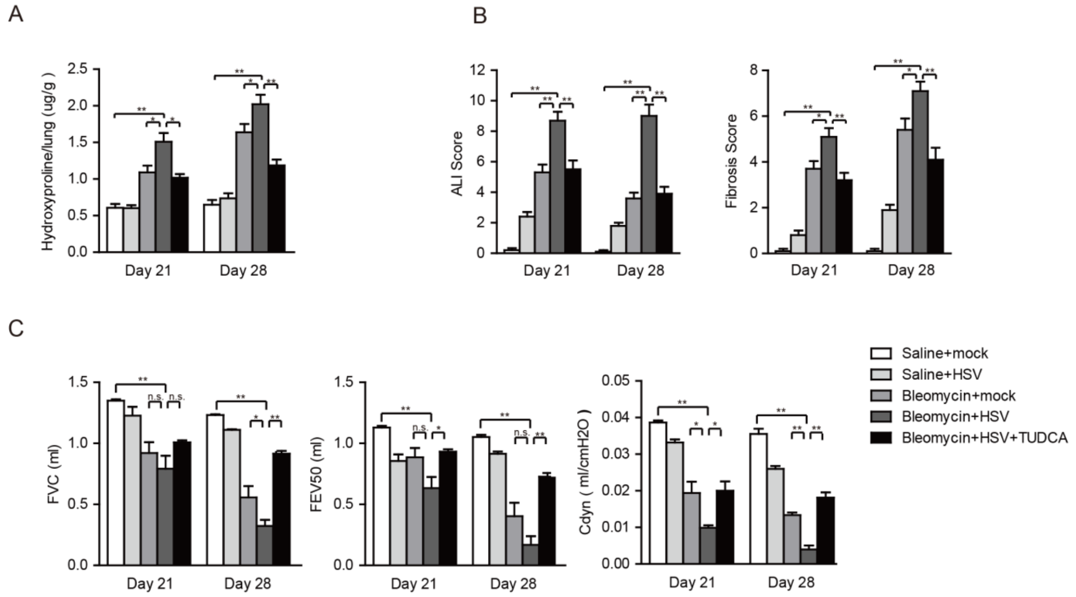

Supplementary Figure S8 HSV-1 infection exacerbated bleomycin-induced lung fibrosis

A. Hydroxyproline content measured by the alkaline hydrolysis method (n=10). **B**. Ashcroft score of acute lung injury and fibrosis on histology (n=10). **C.** Pulmonary function test (n=10). Data are presented as means ± SEM, * *p* <0.05; ** *p* <0.01; n.s. Abbreviation: ALI: acute lung injury; FVC: forced vital volume; FEV50: forced expiratory volume in 50millisecond; Cdyn: dynamic compliance.

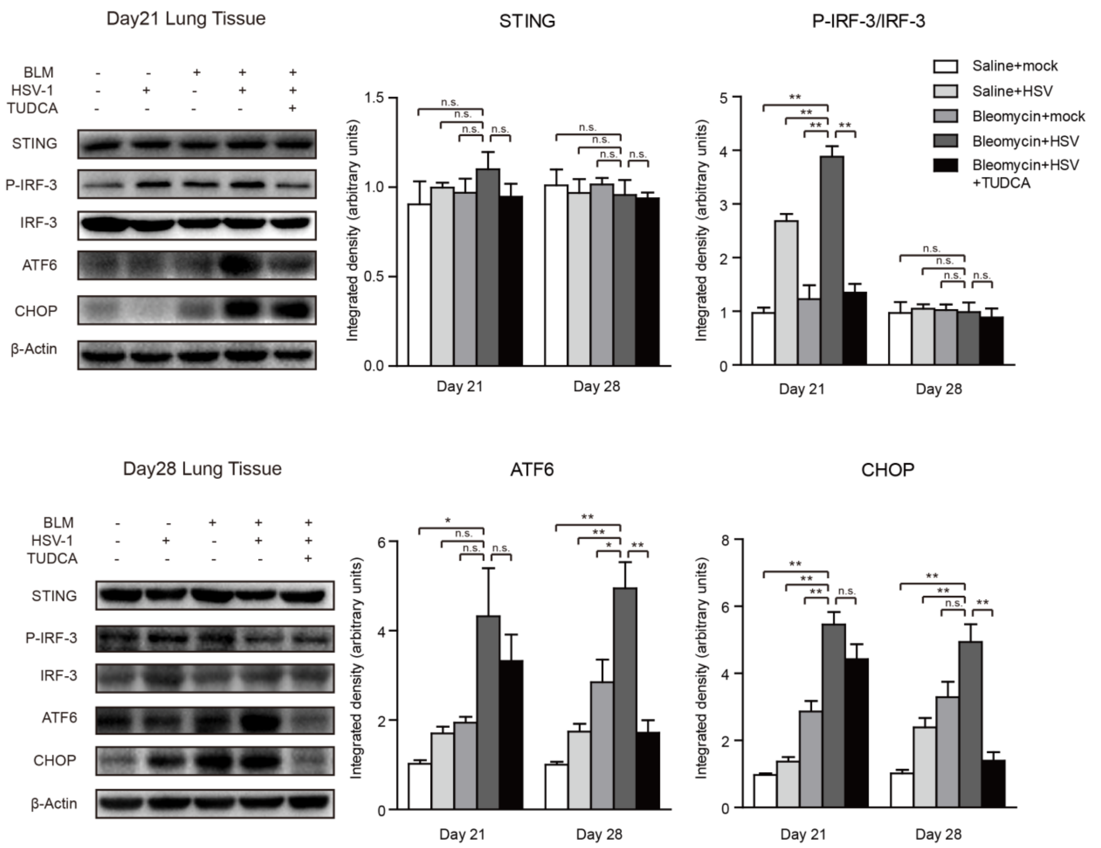

Supplementary Figure S9 HSV-1 infection induced ER stress and down-regulated STING protein expression in mice with bleomycin-induced lung fibrosis

Left: Representative Western blot image of the expression of STING, P-IRF-3, IRF-3, ATF6, and CHOP in the lung tissue of mice on day 21 and day 28 after bleomycin injection. Right: Densitometry analysis of the western blot bands (n=5). The intensity of the protein bands was normalized to β-actin. Data are presented as means ± SEM, * *p* <0.05; ** *p* <0.01; n. s., not significant.

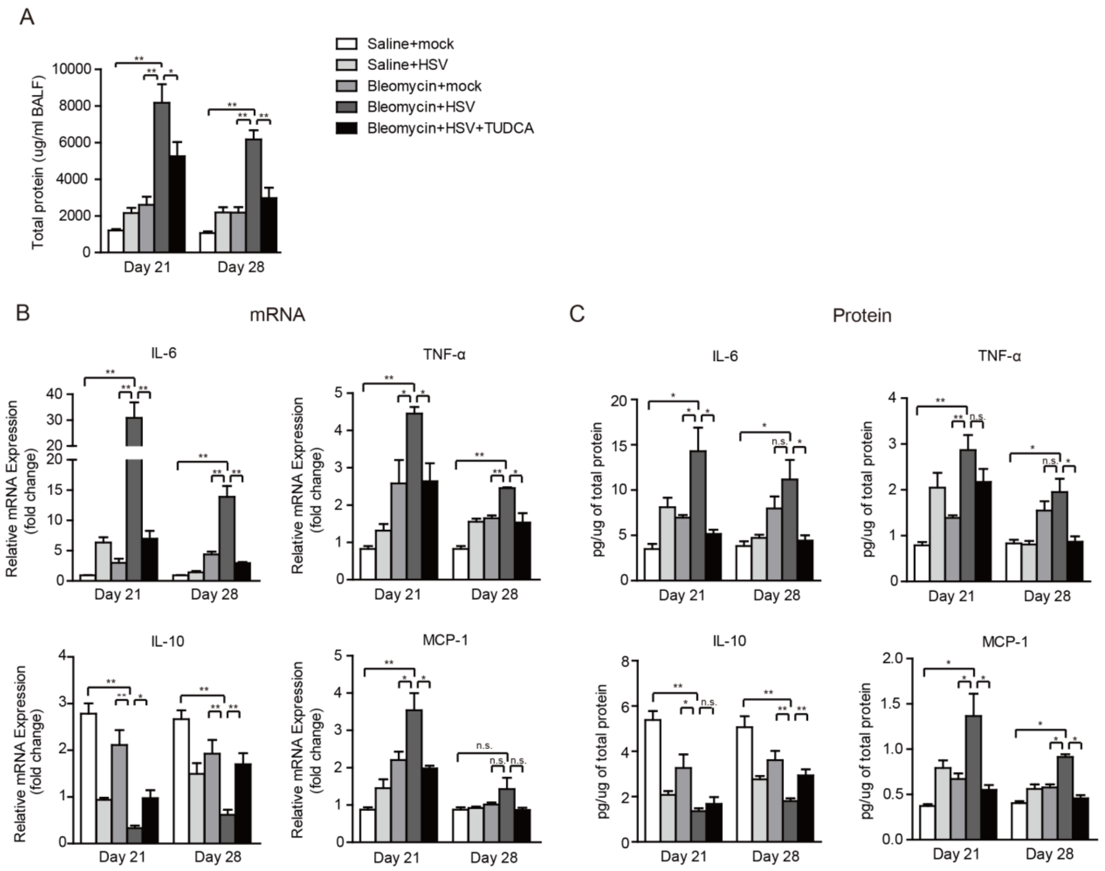

Supplementary Figure S10 HSV-1 infection exacerbated bleomycin-induced lung inflammation response

A. Total protein concentration of bronchoalveolar lavage fluid (BALF) estimated by bicinchoninic acid (BCA) assay (n=10). **B**. mRNA expression of cytokine IL-6, TNF-α, IL-10, and MCP-1 measured by qRT-PCR method (n=10). **C**. Protein expression of cytokine IL-6, TNF-α, IL-10, and MCP-1 determined by ELISA method (n=10). Data are presented as means ± SEM, * *p* <0.05; ** *p* <0.01; n.s., not significant. Abbreviation: BALF: bronchoalveolar lavage fluid; IL-6: interleukin-6; TNF-α: tumor necrosis factor-α; IL-10: interleukin-10; MCP-1: monocyte chemoattractant protein-1.

Supplementary Table S1 Primers used in quantitative real-time PCR assays.

Genes	Species	Forward primers (5'→3')	Reverse primers (5'→3')
STING	Human	CACTTGGATGCTTGCCCTC	GCCACGTTGAAATTCCCTTTTT
IFNα	Human	GCCTCGCCCTTTGCTTTACT	CTGTGGGTCTCAGGGAGATCA
IFNβ	Human	ATGACCAACAAGTGTCTCCTCC	GGAATCCAAGCAAGTTGTAGCTC
CXCL-10	Human	GTGGCATTCAAGGAGTACCTC	TGATGGCCTTCGATTCTGGATT
IL-6	Mouse	TCTATACCACTTCACAAGTCGGA	GAATTGCCATTGCACAACTCTTT
TNF-α	Mouse	CAGGCGGTGCCTATGTCTC	CGATCACCCCGAAGTTCAGTAG
IL-10	Mouse	GCTGGACAACATACTGCTAACC	ATTTCCGATAAGGCTTGGCAA
MCP-1	Mouse	TTAAAAACCTGGATCGGAACCAA	GCATTAGCTTCAGATTTACGGGT
β-Actin	Human	CACCATTGGCAATGAGCGGTTC	AGGTCTTTGCGGATGTCCACGT
GAPDH	Mouse	AGGTCGGTGTGAACGGATTTG	GGGGTCGTTGATGGCAACA

Supplementary Table S2 Microarray raw data

Genes	AE-IPF				IPF				HC			
	AE-IPF1	AE-IPF2	AE-IPF3	AE-IPF4	IPF-1	IPF-2	IPF-3	IPF-4	HC-1	HC-2	HC-3	HC-4
AUTS2	5.08631	4.971507	5.205606	5.146746	5.092468	4.942395	5.203388	5.231975	5.544911	5.631275	5.856902	5.247061
BCL2L1	8.50801	8.182078	8.067672	9.281229	8.615775	8.6038	9.511356	8.670619	9.243556	9.080188	8.846416	9.296376
CCL2	4.217758	3.588587	4.249989	3.904805	3.948853	3.832448	4.050336	4.247602	3.629388	4.648589	3.830635	4.296179
CD40LG	7.013984	5.396449	7.411718	5.992013	7.423977	8.045482	6.520371	6.897446	9.809156	8.494087	8.410874	7.991608
GCH1	8.13027	7.349641	7.813429	7.211213	8.399496	8.700908	7.725816	8.439831	8.289454	7.42184	8.49445	7.944695
HERC6	7.115884	6.534984	6.824279	6.930841	7.002587	7.541759	6.939117	8.170596	7.344502	7.223689	7.178755	6.925665
HLA-DQB1	5.368155	4.82567	5.097763	4.54103	5.561684	4.920125	5.226249	6.600703	5.107155	5.389986	5.190718	5.081264
IFI27	4.729082	4.660765	4.552731	5.317171	4.556016	8.843969	5.007452	5.207821	4.586869	4.685713	5.011302	5.256301
IFIH1	8.302753	6.729865	7.682877	8.356433	8.208922	9.227661	7.493569	9.752732	8.355187	7.814061	8.174386	8.116587
IL18BP	5.779171	5.2881	5.617792	5.275259	5.72057	5.903079	5.636457	5.590582	5.837191	5.530773	6.07427	5.312396
IL32	7.765591	7.774324	7.651758	7.569846	8.246919	8.2228	8.036381	8.50565	8.500691	8.275799	8.028866	7.761539
IRF3	5.543382	5.602776	5.587047	5.55814	5.551014	5.475232	5.69072	5.756001	5.706626	6.029517	5.866644	5.785468
ITGB3	7.526514	8.130913	7.914378	10.12312	8.307283	8.406493	9.392324	9.97623	9.415626	9.751223	8.80628	10.53878
KCTD7	6.574205	6.283323	6.675757	6.195153	7.227352	6.519686	6.706724	6.3409	6.87294	6.629783	6.863297	6.565244
LAMP3	4.471863	4.290314	4.192744	4.670797	4.332139	4.604912	4.354327	4.841499	4.559832	4.568409	4.529748	4.482048
LGALS9	6.931886	7.269657	7.217972	6.83392	6.758427	7.69448	7.006759	7.266114	6.457348	7.252331	7.803111	7.787038
MX1	7.796821	6.732588	7.144514	7.914275	7.699639	8.603591	6.888123	10.53036	8.257975	7.158884	8.038854	7.696961
OA	7.198017	6.048644	6.586237	6.556739	6.94683	9.221851	6.445436	9.119953	6.64228	6.196393	6.986653	7.108394
OAS2	8.509818	6.84837	7.381069	7.581307	7.736256	10.76613	7.446225	11.25326	9.275748	8.470473	9.127057	8.282943
OAS3	6.544044	5.331306	5.51067	6.625189	6.151953	8.608085	5.549637	9.635006	6.776815	6.217839	6.717803	6.789395
STAT2	8.706523	7.854042	8.37369	9.146728	8.530381	9.888062	8.197574	10.3394	8.979796	8.742044	9.314767	8.938851
TCF7L2	5.689598	6.464153	6.546324	5.753688	6.106229	6.966215	5.837729	6.862456	6.171541	5.999005	7.119017	6.958595
TLR3	4.734671	4.38732	4.800263	4.772296	4.810593	4.531466	4.718833	5.002823	4.882485	4.962953	4.989474	4.76946
USP18	5.133997	4.653427	4.838328	4.992215	5.175446	5.645866	4.880591	5.906665	5.176652	5.093799	5.121527	5.076456
